# Access barriers and facilitators to implement mass drugs administration strategies for eliminating trachoma and geohelminthiasis in the department of Amazonas, Colombia

**DOI:** 10.1371/journal.pone.0310143

**Published:** 2024-12-11

**Authors:** Julián Trujillo-Trujillo, Sara Milena Zamora, María Consuelo Bernal Lizarazu, Myriam Leonor Torres Pérez, Olga Esther Bellido Cuéllar, Carol Viviana Araque, Sonia Jaqueline Pulido Martínez, Claudia Marcela Vargas Peláez, Francisco Rossi, Luisa Fernanda Moyano Ariza, Luz Mery Bernal Parra

**Affiliations:** 1 Ministry of Health and Social Protection, Subdirection of Communicable Diseases, Bogotá, Colombia; 2 Universidad Nacional Abierta y a Distancia-UNAD, Escuela de Ciencias de la Salud (ECISA), Bogotá, Colombia; 3 7 Secretaría de Salud de Amazonas, Leticia, Colombia; 4 Fundación Ifarma, Bogotá, Colombia; University of Ghana Noguchi Memorial Institute for Medical Research, GHANA

## Abstract

**Background:**

One of the most important pillars of action to achieve the elimination of trachoma and soil-transmitted helminth infections as a public health problem is the mass administration, at regular intervals, of azithromycin and anthielmintics, respectively, to a high proportion of the eligible population in endemic areas.

**Objective:**

The objective of the study was to identify access barriers and facilitators for achieving coverage goals in the mass drugs administration, azithromycin and albendazole, in the department of Amazonas, Colombia.

**Methodology:**

Implementation research was used, combining three types of qualitative research methodologies to collect information about access barriers and facilitators already described; These were individual and group interviews, focus group discussions and face-to-face intercultural dialogues. We design, validate and apply different instruments with questions adapted to the context and role of the participants, and recorded and transcribed the sessions and analyzed them in qualitative analysis software. We used the Consolidated Framework for Implementation Research (CFIR) to complement the above instrument questions, to guide data analysis, and apply the Consolidated Criteria for Reporting Qualitative Research (COREQ).

**Results:**

Records of 159 participants were included; 21 individual and 3 group interviews, 6 focus group discussions and 4 intercultural dialogues were carried out. 21 strong, 30 weak, 6 neutral barriers as well as 5 weak and 11 strong facilitators were identified. 62% of the strong barriers and 40% of the weak ones were concentrated in the “Outer Setting Domain”. Only 16 facilitators were identified, 44% in the “Innovation” domain.

**Conclusions:**

Multiple political, administrative, geographical, logistical and cultural access barriers, as well as external and internal migration of the population, explain low coverage in mass administration of azithromycin and albendazole. It is necessary to review them individually to implement an improvement plan that also recognizes the identified facilitators.

## Introduction

Trachoma and geohelminthiasis are neglected tropical diseases (NTD) still considered of interest in public health in the Americas region due to their prevalence and high vulnerability of the affected populations [1].

One of the most important pillars of action to achieve their elimination is the mass drug administration (MDA) of azithromycin and albendazole or mebendazole, respectively, which are provided to the entire eligible population of a given geographic area, in order to reduce the prevalence and mitigate or avoid complications associated with those diseases [2]. MDA is indicated in populations where a prevalence of NTD, exceeds the threshold defined by the World Health Organization (WHO) for recognition as a public health problem: Trachomatous inflammation—follicular (TF) ≥5% in children aged 1–9 years [3, 4] or geohelminths ≥20% in children aged 5 to 14 years [5].

MDA for trachoma is framed under component A of the SAFE strategy (Surgery, Antibiotics, Facial cleanliness, and Environmental Measures) [3] and for geohelminthiasis is part of the WHO’s Preventive Anthelminthic Chemotherapy (PCH) strategy [5, 6].

Amazonas is a department located in southern Colombia, in the Amazon rainforest, bordering Brazil and Peru, with an estimated population for 2022 of 82,068 inhabitants, of which 41,360 (50.4%) concentrated in the urban area [7], and the rest distributed in 173 dispersed rural communities. Most of its inhabitants are indigenous people and belong to 26 indigenous ethnic groups [8]. The high population dispersion, cultural and linguistic diversity, lack of roads, high turnover of health workers and the concentration of health services in the capital, added to the high operating costs, represent a great challenge to implement the MDA and public health programs in general.

The Ministry of Health and Social Protection of Colombia, in its role as rector of the public health policy, through different laws, technical guidelines, plans, and programs for communicable diseases has promoted the implementation of MDA strategies to eliminate trachoma and geohelminthiasis as public health problems. It has also defined the source of financing, the access route to drugs, and the roles of the institutions.

The Amazonas Health Secretariate has adopted the national guidelines and subcontracted the public health activities, included MDA for geohelminthiasis, with San Rafael Hospital in Leticia since 2017, which has integrated with the activities of the other public health programs provided to the population, aiming to achieve coverage of at least 75% in each round: twice a year in the preschool and school population with a single dose of mebendazole and albendazole, respectively, considering that the prevalence of geohelminthiasis for the Amazon region in the baseline defined in 2013 was 81.6% [9].

For its part, MDA for trachoma, has been implemented since 2017 directly by the Amazonas Health Secretariate with trained personnel dedicated to the activities inherent to the SAFE strategy, trying to achieve annual coverage of at least 80% in the mass administration of azithromycin.

In both strategies (MDA for trachoma and geohelminthiasis) and at separate times, nursing assistants or public health assistants travel from Leticia to the rural communities by waterway, airway, and/or overland to administer drugs to eligible persons and carry out activities inherent to these programs, following a work plan designed by the Amazonas Health Secretariate, and which in the case of geohelminthiasis has been executed in conjunction with other public health actions.

The estimates coverage for azithromycin have been carried out using as the source of the numerator and denominator a nominal database, parameterized by the ministry of health.

Despite the above, coverage targets reported by Ministry of Health have not been met in any round nor any of the programs: Azithromycin: 30% in 2015, 45% in 2016, 51% in 2017, 51% in 2018, 57% in 2019, and 21% in 2020; Albendazole 52% in 2016, 70% in 2017, 40% in 2018, 50% in 2019, and 55.9% in 2021 with negative economic consequences for the health system and the possibility of generating resistance to these drugs [10–12].

We consider that these low coverages can be explained by the migration of the population, the late contracting of actions, the multiplicity of tasks assigned to health workers, the lack of supplies, the lack of community participation, a deficient information system and differences between the real population and that estimated by the National Administrative Department of Statistics (DANE), which affects coverage because it is the official denominator for the calculation. Other difficulties are due to structural problems in the Colombian health system, which are particularly acute in the Amazon, such as the absence of service delivery networks and permanent health workers in rural communities.

This study was developed to identify access barriers and facilitators to implement MDA for trachoma and geohelminthiasis in the department of Amazonas, in order to adapt national guidelines and local micro-planning, based on the findings.

## Methods

### Study design

We use Implementation Research to analyze contextual aspects that influence mass drug administration; three types of qualitative research methodologies based on phenomenological theory [13] were used to collect information on access barriers and facilitators to implementing MDA strategies for trachoma and geohelminthiasis, these were individual [14] and group interviews [15], focus group discussions (FGD) [16], and face-to-face intercultural dialogues [17], where possible; group methods were applied when several participants shared the same role. The selection of one or another method depended on the number of participants in the sessions.

An anthropologist, MSc Gender and a health professional with a master’s degree in epidemiology let the qualitative methodologies, both attached to the ministry of health and with experience in qualitative research, a background of working in indigenous communities, and knowledge of the SAFE and PCH strategies. Two health professionals from the Amazonas Health Secretariate, previously trained in the application of the study instruments supported the development of some individual interviews with the strategies beneficiaries.

### Study population

Key actors from the health sector at the national, departmental, municipal, and institutional levels with managerial, administrative, coordination, supervision, and implementation roles; from the education sector in the roles of coordinator or teacher; community actors with zonal influence with the role of leaders, namely, indigenous governors and others with local influence; in the communities, an agent of ancestral medicine; and indigenous and multiracial beneficiaries of the MDA of azithromycin and albendazole were identified and included.

The place of residence of the participants included the department of Amazonas, municipalities of Leticia and Puerto Nariño, seven rural communities where MDA had previously been implemented, and Bogota for participants from national institutions.

### Sample size and sampling

Given the diversity of roles involved, various sampling strategies were defined. The initial sample frame of stakeholders from the health sector institutions that met the inclusion criteria was 62 individuals. Of these, 18 held key and unique positions within the institutions, with managerial and coordinating roles, and were included in their entirety. The remaining 44 individuals had an implementer role and were selected randomly from separate lists of men and women to ensure representativeness of both sexes and according to time of experience in MDA (greater or less than one year).

Moreover, the most prominent and representative school in the rural and urban areas was selected to interview the teachers; the latter were selected randomly for the FGD from lists separated by sex among those who had participated in previous rounds of MDA. The number of FGD was unlimited until, in the opinion of the researchers, data saturation had been reached. Table 1 represents the different participants and applied methodologies.

**Table 1:** Participants and methodologies applied to collect the information.

Stakeholders	Role of the participants	Municipalities or communities represented	Individual interviews			Group interviews				Focus group discussion				Intercultural dialogue				Total participants by sex		
			Female	Male	Total	Female	Male	Total	Number of groups	Female	Male	Total	Number of focus groups	Female	Male	Total	Number of sessions	Female	Male	Total
Ministry of Health and Social Protection*	Directors of the Ministry of Health	Bogota	**1**	** **	**1**	** **	** **	** **	** **	** **	** **	** **	** **	** **	** **	** **	** **	**1**	**0**	**1**
	Technicians of the Ministry of Health		**1**	**1**	**2**		** **		** **		** **		** **		** **		** **	**1**	**1**	**2**
Amazonas Health Secretariat*	Directors	Leticia	**3**		**3**	** **	** **	** **	** **	** **	** **	** **	** **	** **	** **	** **	** **	**3**	**0**	**3**
	Trachoma and STH programs professionals	Leticia	**2**	** **	**2**	**2**	** **	**2**	**1**	** **	** **	** **	** **	** **	** **	** **	** **	**4**	**0**	**4**
	Trachoma program field health workers (technicians)	Leticia	** **		** **		** **		** **	**5**	**6**	**11**	**2**		** **		** **	**5**	**6**	**11**
Puerto Nariño Health Secretariat*	Technicians from Puerto Nariño Health Secretariat	Puerto Nariño	** **	** **	** **	** **	** **	** **	** **	**4**	**2**	**6**	**1**	** **	** **	** **	** **	**4**	**2**	**6**
San Rafael de Leticia Hospital*	Directors of the territory’s hospitals	Leticia	** **	** **	** **	** **	**2**	**2**	**1**	** **	** **	** **	** **	** **	** **	** **	** **	**0**	**2**	**2**
	Technicians from San Rafael Hospital	Leticia	** **	**1**	**1**	**2**	** **	**2**	**1**	** **	** **	** **	** **	** **	** **	** **	** **	**2**	**1**	**3**
San Juan Bosco educational institution	Teachers	Leticia, Km 7	** **	** **	** **	** **	** **	** **	** **	**5**	**4**	**9**	**2**	** **	** **	** **	** **	**5**	**4**	**9**
Indigenous leaders	Governors, captains, indigenous leaders.	Puerto Santander	** **	**1**	**1**	** **	** **	** **	** **	** **	** **	** **	** **	** **	** **	** **	** **	**0**	**1**	**1**
		Puerto Nariño	**1**	** **	**1**	** **	** **	** **	** **	** **	** **	** **	** **	** **	** **	** **	** **	**1**	**0**	**1**
		Rural community 1	** **	** **	** **	** **	** **	** **	** **	** **	** **	** **	** **	**26**	**76**	**102**	**1**	**26**	**76**	**102**
		Rural community 2	** **	** **	** **	** **	** **	** **	** **	** **	** **	** **	** **				**1**			
		Rural community 3	** **	** **	** **	** **	** **	** **	** **	** **	** **	** **	** **				**1**			
		Rural community 4	** **	** **	** **	** **	** **	** **	** **	** **	** **	** **	** **				**1**			
Ancient medicine agents	Agents of ancestral medicine (midwife)	Puerto Nariño	**1**	** **	**1**	** **	** **	** **	** **	** **	** **	** **	** **	** **	** **	** **	** **	**1**	**0**	**1**
Beneficiaries of MDA strategies	Indigenous	Puerto Nariño	** **	** **	** **	** **	** **	** **	** **	**2**	**2**	**4**	**1**	** **	** **	** **	** **	**2**	**2**	**4**
		Comunidad Yoi, Km 5	**4**	** **	**4**	** **	** **	** **	** **	** **	** **	** **	** **	** **	** **	** **	** **	**4**	**0**	**4**
	Mestizos	Nienemechi 1 to 5	**5**	** **	**5**	** **	** **	** **	** **	** **	** **	** **	** **	** **	** **	** **	** **	**5**	**0**	**5**
**Total**			**18**	**3**	**21**	4	**2**	**6**	**3**	**16**	**14**	**30**	**6**	**26**	**76**	**102**	**4**	**64**	**95**	**159**

*health sector participants

^a^ *Female*

^b^ *Male*.

For the inclusion of the beneficiaries of the strategies, initially, three rural communities were randomly selected and visited within the framework of the MDA; a minimum number of four participants were included in each of them, considering that a maximum of 12 participants was expected to data saturation [18]. On the other hand, the 104 indigenous peoples with the role of community leaders or indigenous governors represented 100% of the rural communities and were included in their totality.

Finally, due to the lack of a list of traditional medicine agents and the communication difficulties, a representative of the agents of ancestral medicine, referred by the public health technical team of the municipality of Puerto Nariño, was intentionally selected.

### Inclusion and exclusion criteria

The inclusion criteria were: being older than 18 years or having parental consent; being currently linked or have been linked in a previous year to any public institution in the health sector at the levels and with the roles described; being aware or having participated directly or indirectly in the implementation of the SAFE or PCH strategies; belong to an educational institution where one or both strategies have been implemented, or be a zonal or community leader, or agent of traditional medicine recognized by the community, or be indigenous or multiracial beneficiary of one or both strategies, speak Spanish; and express willingness to participate in the study.

The exclusion criterion for institutional participants was to have less than six months or no experience in trachoma or geohelminthiasis programs. For the community or non-institutional participants, the exclusion criterion was to have never participated as a beneficiary or collaborator in any MDA activities.

### Design of data collection tools

Seven types of instruments were designed to guide the interviews, focus groups, or intercultural dialogues with questions adapted to the language, educational level, and role of the participants, grouped in the following domains: demographic information of the respondents, general knowledge of the trachoma and geohelminthiasis programs, resources for the programs operation, MDA planning and development, information and analysis system, feedback of the process, and perception of stakeholders to improve the process.

An instrument was applied to each stakeholder according to their role, as follows: 1) Executives of the Ministry of Health and Social Protection, the departmental or municipal departments of health, and of the hospital; 2) Trachoma or geohelminthiasis professionals and technicians of the same institutions; 3) Teachers; 4) Representatives of the MDA beneficiaries, such as leaders, indigenous governors; 5) Representative or agent of ancestral medicine; 6) Indigenous peoples beneficiaries and; 7) Multiracial beneficiaries.

The domains and constructs from the updated Consolidated Framework for Implementation Research (CFIR) [19] were used to include questions related to constructs and domains not initially contemplated and to guide the systematic evaluation of the context in which MDA was implemented. Before applying the instruments, they were validated to adjust the language and verify the questions’ adjustment to the participants’ context and role.

### Application of qualitative techniques of data collection

The qualitative research methodologies were conducted after identifying and inviting key actors to whom the study was socialized; the data collection began on May 2, 2022 and ended on October 31 of the same year. The following methodologies were applied in person in different sessions for the participants of each role as follows:

Individual and group interviews: They were used when, for logistical reasons, it was impossible to gather more than two participants with the same role or, in the case of the management, coordination, or other roles, when they were unique.

Focus group discussions: Separate FGDs were formed for institutional actors not interviewed individually who shared the same role, trying, when possible, to conduct two groups per stratum or role to achieve a more complete understanding of the phenomenon under study [20] and mixing men and women to ensure representativeness of both sexes and improve the quality of the discussion.

The groups were formed as follows: three in the Amazonas Health Secretariate, one for people with coordination or management support functions for the trachoma program, two for those responsible for implementing the MDA (one for old and one for new beneficiaries with at least six to 12 months of experience); two more FGDs in the San Rafael Hospital in Leticia, one for managers and one for nurses implementing the MDA. In the Municipal Department of Health for Puerto Nariño, an FGD was created for the implementers of the public health programs; two FGDs were created for teachers at the San Juan Bosco school (IESJB for its Spanish acronym) in Leticia. Between three and six people participated in each group [20]. The methodologies were applied in their workplace for institutional actors and at home for the rest of the participants.

Two professionals with the described profiles conducted the focus groups, one as moderator and the other as an observer, which was rotated; the observer took notes during the development of the FGDs. At least two members of the Secretariat of Health of Amazonas who were not part of the research group accompanied each FGD and intercultural dialogue; given that they were the people recognized by the community, they were responsible of guiding the tours and introducing the interviewers.

For the institutional actors, surveys at the workplace were applied, and for the others at the residence. In order to apply all the methodologies, an effort was made to generate a warm and trusting environment that allowed sincere and fluid communication with the participants. The instrument designed to guide the questions according to the role of the participants was used, and the necessary counter-questions were asked until data saturation was achieved according to the moderator’s criteria. The average duration of the interviews was one hour and two hours for the focus groups, and each intercultural dialogue lasted approximately four to five hours.

All sessions were recorded and later transcribed to Microsoft Word by technicians from the Amazonas Health Secretariate. The quality of the transcription was verified and adjusted from the audio recordings, as necessary, by the two researchers who performed the coding. The individual and group interviews and FGDs were conducted in Spanish, and people of the Murui (Huitoto), Muiname, Bora, Miraña, Yukuna, Tanimuka, Letuama, Karijona, Makuna, and Andoque indigenous peoples participated.

### Information processing

The qualitative data analysis software ATLAS.ti, version 9 from Berlin, Germany, was used to code the transcribed texts of each of the applied methodologies by two researchers; the above, based on the constructs predefined by the researchers.

Those constructs were adjusted in their wording according to need, and on the emerging constructs to generate the reports that allowed extracting quotes, as well as the barriers and facilitators of the MDA, with which a coding tree was made (S1 Fig).

The updated CFIR domains, constructs, and sub-constructs and their definitions were used as a structure to relate the access barriers and facilitators identified in the study and as a theoretical framework to guide the systematic assessment of the context in which the MDA was being applied. Similarly, the rules of this conceptual framework were used to determine the positive (facilitators) or negative (barriers) valence and its scale to rate the degree of influence on the implementation (scales -2,-1, 0, +1, and +2) [21]; this rating was carried out independently for each construct and sub-construct by three researchers, one of national level and two of the departmental level. The final value assigned was the one that was most repeated (S1 Table). When differences of more than one were observed on the scale of the individual ratings, the final value was determined by consensus.

The results found in the study were validated by the institutional participants who were still working in the institutions, and the final results were represented in a map or heat table to facilitate their interpretation.

Finally, to complement the reporting of the work, the Consolidated Criteria for Reporting Qualitative Research (COREQ) were used [22]. (S2 Table).

### Ethical aspects

The Research Ethics Committee of the Universidad Nacional Abierta y a Distancia (UNAD) approved the study by Act number 400.115 of June 8, 2021. Participation in the study was voluntary, and all participants signed a written consent after the presentation of the researchers and socialization of the study objectives.

## Results

A total of eight institutions or stakeholders in the MDA were identified and included in the study; four of them belonged to the health sector, which were the Ministry of Health and Social Protection, Amazonas Health Secretariate, Municipal Health Secretariate for Puerto Nariño, and the State-Owned Social Welfare Enterprise (ESE for its Spanish acronym), San Rafael Hospital of Leticia. Of the other four, one was from the education sector and the remaining three from the community, as follows: San Juan Bosco School, indigenous leaders, an agent of ancestral medicine (midwife), and indigenous peoples and multiracial beneficiaries of the MDA of azithromycin and albendazole. In the study participated 159 people, 64 women and 95 men, representing 11 different localities, ten in the department of Amazonas and one in Bogota (the participants from the ministry of health); 21 individual interviews, 3 group interviews, 6 FGDs, and 4 intercultural dialogues were conducted. All participants who met inclusion criteria participated in the study. Table 1 shows the sampling, the main characteristics of the participants, their number, and the methodologies applied.

**Fig 1:**
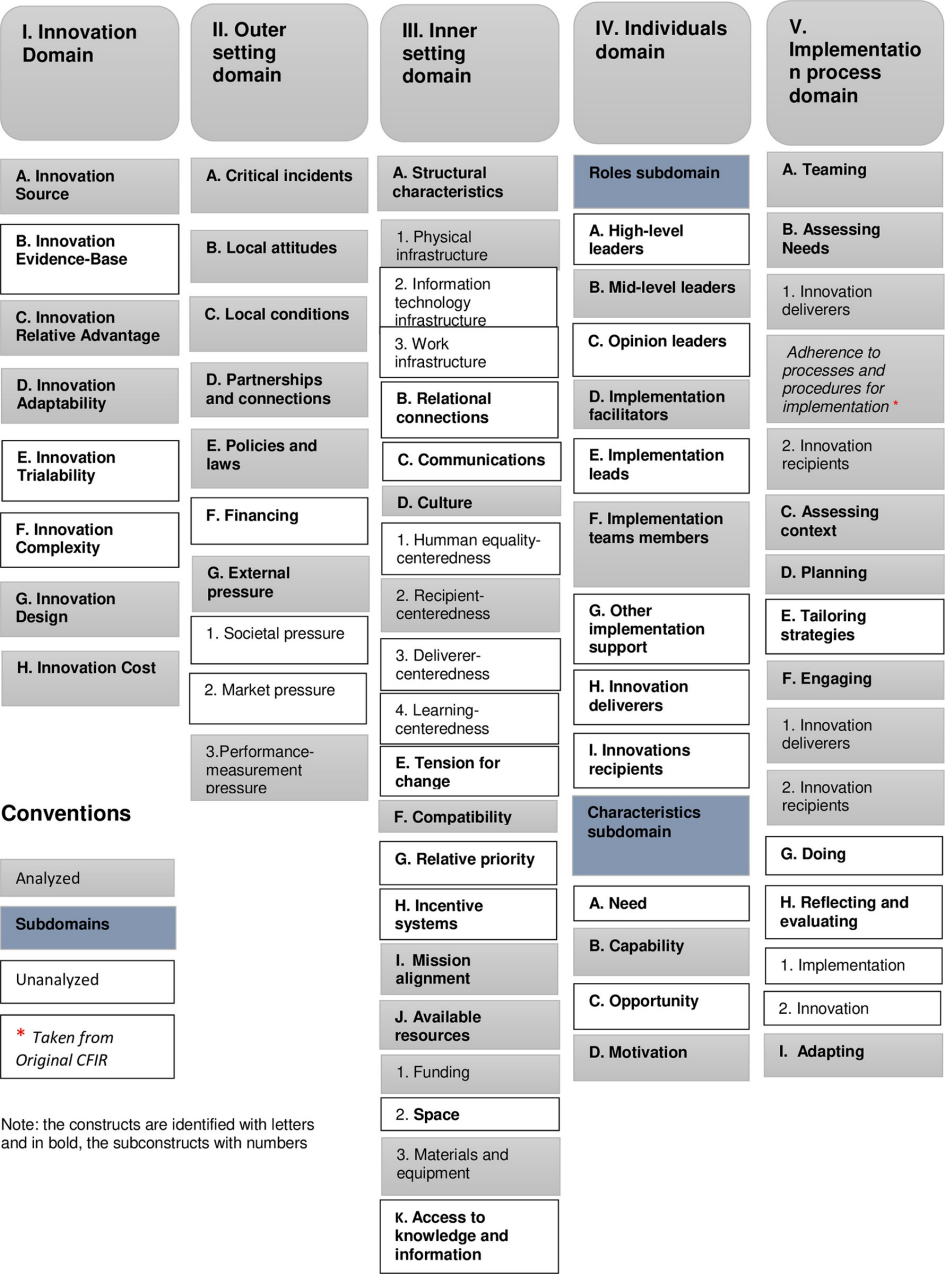
Domains and constructs of the updated CFIR framework, analyzed in the study.

The five domains of the CFIR, two sub-domains, 27 constructs, and 9 sub constructs of its updated version were analyzed. As follows: Five constructs in the Innovation domain, five in the External Environment Configuration domain, five in the Internal Environment domain, five in the Individuals domain, and six in the Implementation Process domain. An additional sub construct from this conceptual framework’s original version was included, but it was not related to those in the new version. Fig 1 shows details of the domains, constructs, and sub constructs analyzed.

### Innovation domain

Five CFIR constructs were analyzed in this domain; one strong barrier to access, six weak, two neutral, as well as one weak and six strong facilitators were identified.

In the "Innovation Source" construct, a loss of trust was perceived in some communities toward the institutions due to non-compliance with community care agendas, negative experiences in previous rounds of MDA, and a questioning of the quality of the service offered by the health service provider, not necessarily related to MDA but impacted the perception of the services negatively.

*"…When deworming in the communities, we always tell them that we will go twice a year; thus, we go the first time, and they are left waiting for the second time. We lose credibility because they feel that we are lying to them; they feel deceived*." [Translated quote from its original in Spanish] B1-P6, SSA Implementer

Regarding the "Innovation Relative Advantage" construct, the positive perception of the benefits of deworming with anthelmintic (albendazole) prevailed, and the pleasant taste of this drug was recognized as a facilitator. Most participants reported that deworming with albendazole was more accepted than deworming with medicinal plants, although this practice persisted.

*"The population was very open and willing for these interventions, even though we carried only purgative for children; thus, adults also wanted to be dewormed."* [Translated quote from its original in Spanish] B2-P53, HSRL Implementer

*"Some people in the community are afraid of doctors*, *hospitals; they prefer medicinal plants and the treatment of witches; they only go to the doctor when that treatment does not work for them*.*"* [Translated quote from its original in Spanish] B3-P61, Teacher of IESJB

On the other hand, indigenous participants recognized a limitation of ancestral medicine to treat trachoma; therefore, azithromycin was generally well accepted.

*"… We have it very clear, when it is not possible to provide treatment from the cultural point of view, one must access Western medicine, and when Western medicine cannot be applied, we have to do it with our medicine; indeed, elders here know it, we have it very clear because, sincerely, there are diseases that have no cure or cultural treatment."* [Translated quote from its original in Spanish] B4-P50, Indigenous Governor

The delivery of drugs was identified as "tangible," which made the community feel "seen," not just as the recipient of educational talks.

Regarding the "Innovation Adaptability" construct, it was identified that the MDA was carried out after consultation processes with the leaders of zonal and local indigenous peoples organizations, and in its implementation, it was integrated with strategies of health information and education and communication for health. The personnel who carried out the drug distribution had a clear need to adjust the interventions to the local culture and context; the improvements in these sociocultural adaptations were evident in trachoma but not so much in geohelminthiasis.

"*We have seen it, and we have learned it; one must understand that not everyone speaks Spanish; I must understand that what is important to me is not important to others; one must talk much more about sanitation; I cannot go to your homes and tell you that you must do this and that, I must listen. I have another type of strategy to interact and reach intermediate points, and I think we still have a long way to go*." [Translated quote from its original in Spanish] B5-P55 SSA, STH

Regarding the “Innovation Design” construct, the only strong barrier mentioned was the impossibility of accessing a population census to determine real MDA coverage. In this regard, it was stated that the official data was overestimated and the multiplicity of censuses of the different programs and sectors did not agree with each other; therefore, the coverage calculations were considered underestimated.

Even though the MDA for trachoma in the department of Amazonas was designed to be implemented independently from other public health programs, which was positively valued by the health team, the beneficiaries’ perception of a lack of comprehensiveness to address the multiple health needs of the population was identified as a barrier to access, however, this is mainly because they felt that they were neglected in their other health aspects.

**Fig 2:**
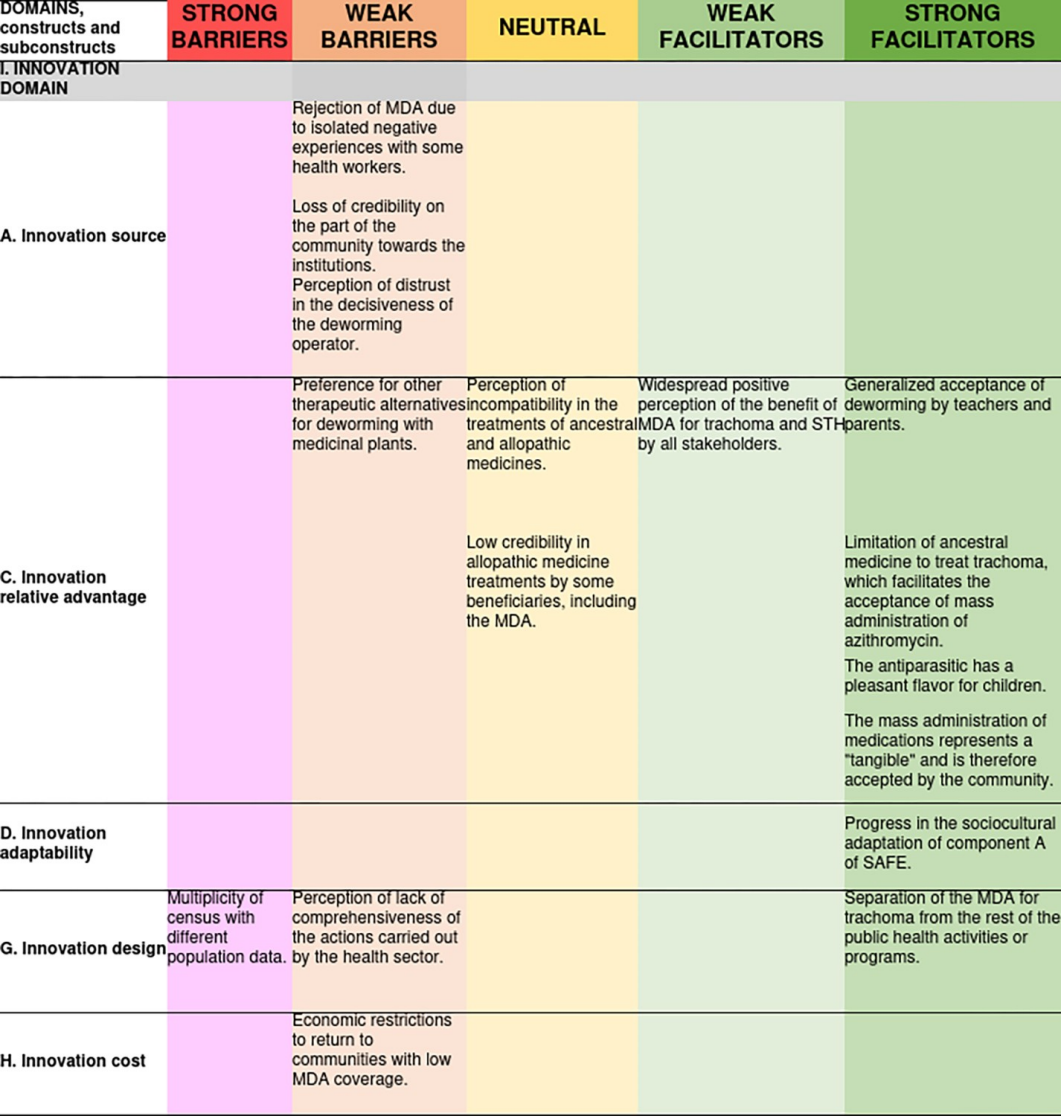
Access barriers and facilitators related to the innovation domain and its degree of influence on the MDA for trachoma and geohelminthiasis.

Finally, in the "Innovation Cost" construct, it was identified that participant from health institutions perceived MDA as very expensive, given the geographic characteristics and communication routes. They mentioned that the budget allocated was insufficient to visit communities with low coverage a second time or for the MDA team to stay longer in the communities. It was also noted that no coordinated work was carried out with the health promoters in some communities to treat people who were absent during the MDA rounds; likewise, no fixed distribution points had been considered part of the strategy design. Fig 2 presents the specific barriers and facilitators of the Innovation domain and represents their valence in a heat graph.

### Outer setting domain

Five constructs and one sub construct of the CFIR were analyzed in this domain; 13 strong and 12 weak access barriers and no facilitators for MDA were identified. The overall domain rating was considered critical.

Concerning the "Critical Incidents" construct, unforeseeable or beyond the control of MDA planning circumstances or barriers were identified in which implementing an innovation would be too costly. They were the high mobility of the population due to subsistence activities, external migration to neighboring countries (Peru and Brazil) for job opportunities, incapacity due to sickness of some health workers who carried out the MDA due to disease during fieldwork, lack of fuel in the local market to mobilize, areas banned due to the presence of illegal armed groups, and the occurrence of mild or moderate adverse reactions to drugs in previous rounds, among others.

When analyzing the "Local Attitudes" construct, it was evident that some indigenous leaders were dissatisfied with the health services offered to the population and did not differentiate the individual provision of services from the MDA rounds, making the development of the latter difficult. In addition, the negative influence of patriarchal cultural patterns that affected coverage was recognized since some women who were part of the MDA teams were sometimes not heard, and their recommendations may have had less impact.

Regarding the “Local Conditions” construct, participants from health institutions (Table 1) reported the limited availability of local health personnel and the presence of geographic and climatic barriers limiting access to communities for MDA, which added to the high dispersion of the population.

*“In Caqueta, the problem is that you have to pass the waterfalls from Araracuara to get to Puerto Santander and then continue upstream… you have to go along the jungle path from Puerto Santander to Puerto Arturo and then to Angostura.”* [Translated quote from its original in Spanish] B6-P26 Dir. of ESEHSRL

*"*…*it is a very difficult situation because the communities are sometimes located along creeks*, *so it is difficult to enter there during the low water season making it impossible to assist these communities*. *This happened to us last year during the medical brigades…"* [Translated quote from its original in Spanish] B7-51 Dir. of ESEHSRL

The analysis of the “Partnerships and Connections” construct made it possible to identify the lack of coordination between institutions at the national and local levels and indigenous authorities, which led to a crossover of activities that affected MDA coverage.

In the “Policies and Laws” construct, it was reported that political aspects and decisions of this order negatively influenced the opportunity to hire human talent and delayed the start of the MDA on some occasions for the benefit and safety of health workers.

*“…due to the presidential election days also cause that there are different things in rural areas because of the illegal groups, we decided not to go out until after July, when it will be calmer.”* [Translated quote from its original in Spanish] B8-P5 Dir. of SDSA.

The obligation to outsource MDA to third parties and the limitation of the commission time of the personnel who carried out the implementation were strong barriers stated by the participants.

**Fig 3:**
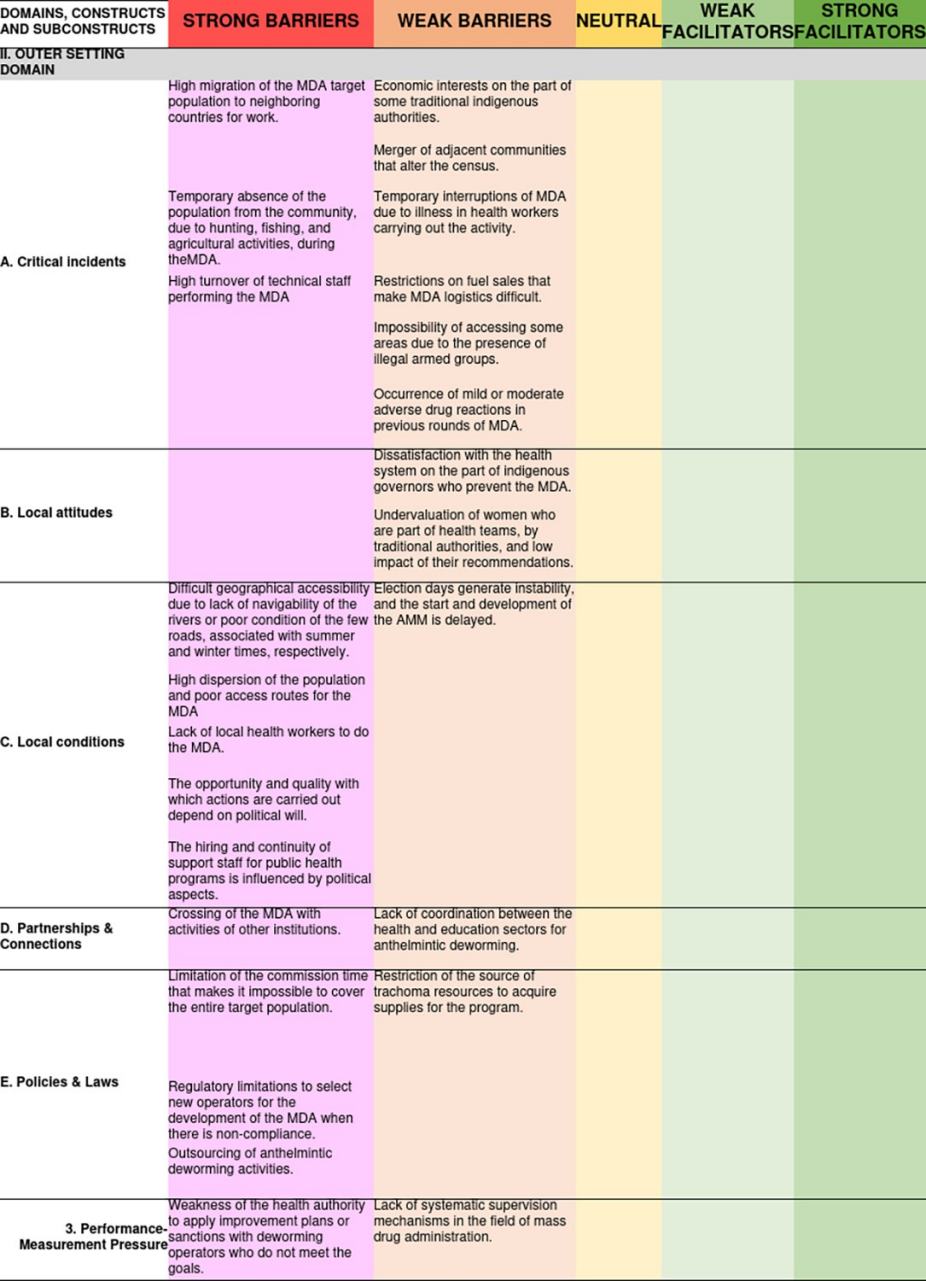
Access barriers and facilitators related to the configuration of the outer setting domain and its degree of influence on MDA for trachoma and geohelminthiasis.

On the other hand, in the “Performance Measurement Pressure” sub construct, it was identified that no supervision mechanisms had been implemented during the MDA for trachoma, arguing high costs, and it was initiated in the last year for the case of geohelminthiasis. Additionally, the local health authority’s weakness in supervising the deworming operator and applying improvement plans for non-compliance with the coverage goals was identified. Fig 3 shows the barriers to access and facilitators of MDA related to the configuration of the external environment, its valence, and its degree of strength.

### Inner setting domain

In this domain, five constructs and four sub constructs were analyzed; three weak barriers for MDA and no strong barriers were identified, and two neutrals and two strong facilitators were identified.

In the "Structural Characteristics" construct, the "Physical Infrastructure" subconstruct was analyzed; it was a negative perception among some participants that the hospital executing the actions did not have the infrastructure capabilities for the transportation logistics of the personnel that carried out the MDA (boats, motors, vehicles) to cover the entire territory or to do it on time. Therefore, the criterion of capabilities could not be a competitive advantage to choose it as operator of the MDA but was valued neutrally because the public health resources allocated to the hospital when contracted allowed subcontracting such infrastructure.

Within the "Culture" construct, the "Recipient-Centeredness” sub construct was analyzed; some team members of the MDA and indigenous beneficiaries perceived that azithromycin or albendazole could not be taken after consuming some local food or beverages; therefore, they rejected the medication.

*"…is that this is the custom…all the houses had açaí, they said that this fruit is contraindicated with medicines, it is like their belief, it is breakfast, dinner, and even lunch, so they prefer not to take the drug to be able to eat açaí…"* [Translated quote from its original in Spanish] B9—P47 New trachoma implementers.

In the "Compatibility" construct, it was identified that since the MDA demands 100% coverage of rural communities, it was necessary to start early, but it could not be planned nor contracted independently of the rest of the public health activities that the hospital must carry out; these activities were usually contracted in the second half of the year.

*“…we depend on a longer administrative process since there is not just one trachoma component but rather several, chronic transmissible diseases, mental health, sexual health, etc. These are the components that go into the contracting package or matrix that is made with the hospital…”* B10 –P2 Dir. of SDSA

Regarding the "Mission Alignment" construct, the local public health agenda included geohelminthiasis and trachoma as one of the priorities of the local government, which facilitated the consolidation of those programs that had MDA as one of the pillars of action.

**Fig 4:**
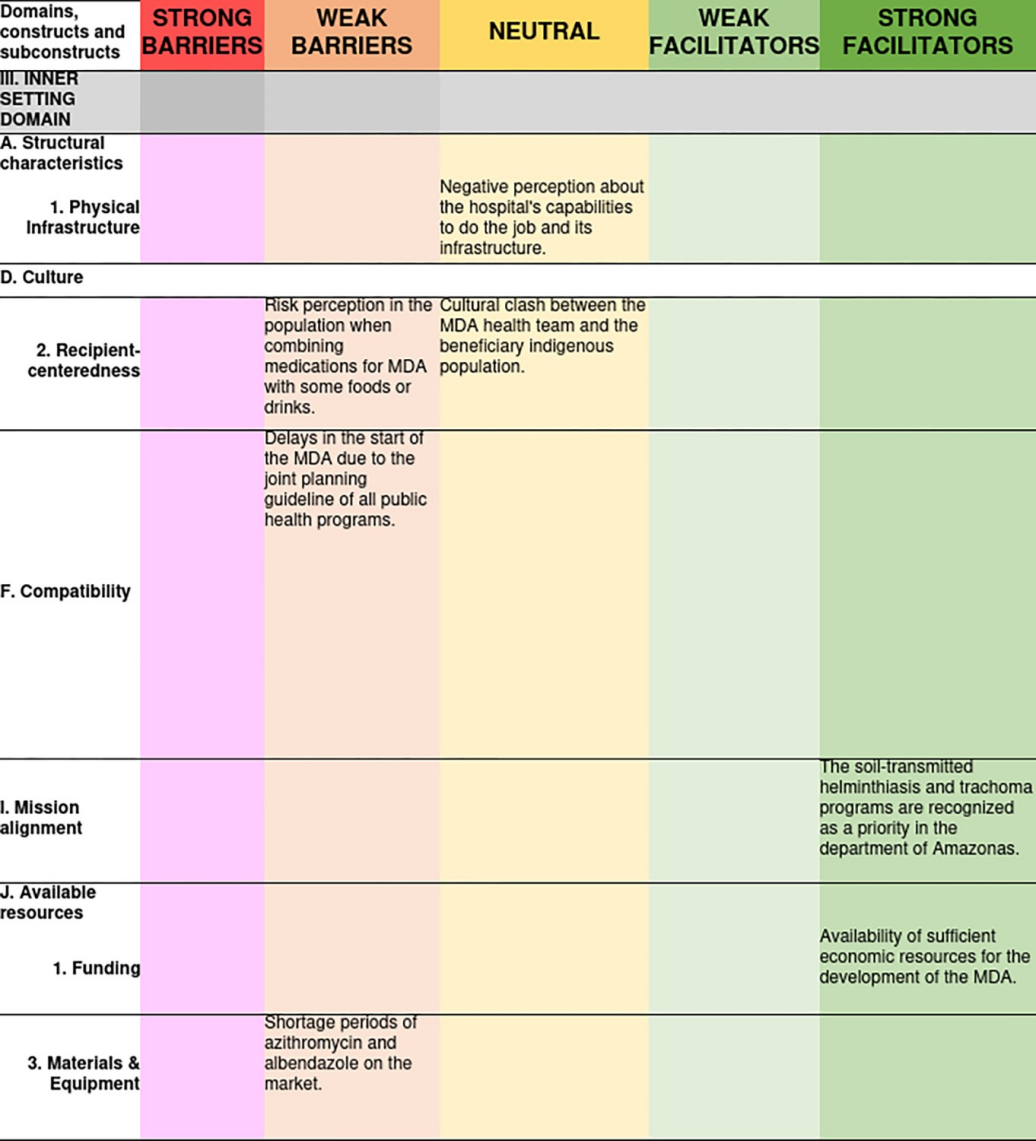
Access barriers and facilitators related to the inner setting domain and its degree of influence on MDA for trachoma and geohelminthiasis.

Within the “Available Resources” construct, the “Funding” and “Materials and Equipment” sub constructs were analyzed; in the first, it was stated that the prioritization of trachoma and geohelminthiasis in the local public health agenda enabled access to resources for the deployment of azithromycin and albendazole MDA, which was considered a strong facilitator. In the “Materials and Equipment” sub construct: during the year 2022, probably as an effect of the COVID-19 pandemic that was ending, there were difficulties in purchasing azithromycin in the dosage form of suspension and albendazole to deworm people older than 15 years, i.e., those outside the population targeted by the donation. In Fig 4, see specific barriers and facilitators and their degree of influence on MDA coverage.

### Individuals domain

Five CFIR constructs were analyzed within this domain; one specific access barrier to MDA was rated as strong, three weak three facilitators as weak, and three facilitators as strong. The results for each construct are shown below.

“Mid-level leaders” construct, institutional mid-level leaders and coordinators of trachoma and geohelminthiasis programs had the autonomy to plan MDA activities but linked to other public health programs. Likewise, indigenous zonal leaders actively participated during the consultation processes with the representatives of the institutions, and most of them recognized its benefits.

“Implementation Facilitators” construct: Some health workers who carried out the MDA had several years of experience with this and other programs; they performed well in rural areas and were recognized and accepted by the people benefiting from these strategies.

The “Implementation Team Members” construct: It was identified that hiring indigenous health workers from rural communities, speakers of native languages, and accepted by the community was a strength to carry out the MDA in the department of Amazonas. Likewise, the hiring of indigenous people from the community in the role of “facilitators,” locally called “ethnic liaisons”, to communicate the decisions of these consultation processes to the local authorities, to mediate between the technical teams and the communities, and encourage their participation in the MDA for trachoma and geohelminthiasis and in the other actions of the public health programs was identified as a facilitator.

“Capability” construct, although the health team that carried out the MDA included people with “Facilitators” or “ethnic liaisons” roles, selected by the indigenous leaders to coordinate this activity in each community, it is mentioned that they, as the rest of the team members, come from Leticia. They could not give the community prior notice about the MDA’s development and the date it would be carried out. It was argued that this caused the population to continue its normal dynamics, characterized by a marked temporary absenteeism resulting in low MDA coverage.

*“…the hospital carries out a consultation process with the indigenous organizations and hires ethnic liaisons, which we have requested as part of the collective intervention plan. These ethnic liaisons should be responsible for letting the community know that the health commissions are going to arrive, that there are going to be different health actions. They are the spokespersons between the community and the health personnel. However, when we get to the communities, they state that they did not know that the Collective Intervention Plan was coming and that they were not expecting us on those dates…”* B11- P11 Coo SDSASTH

Regarding the “Motivation” construct, some participants (Beneficiaries of MDA strategies and indigenous leaders) perceived a passive role in some members of the health teams, especially those who simultaneously carried out MDA and other activities of other public health programs, and a lack of sense of belonging, which negatively impacted MDA coverage.

“*This outsourcing has some administrative situations that make a more timely contracting difficult. That third party carrying it out, how much does it really develop it in the way it is stipulated or as it was defined? If it is someone temporary in this program, surely, he/she is attending to another issue, doing an activity carelessly… thus, I think outsourcing or contracting with a third party has not worked so well for us”* B12-P12 SSA Technician.

**Fig 5:**
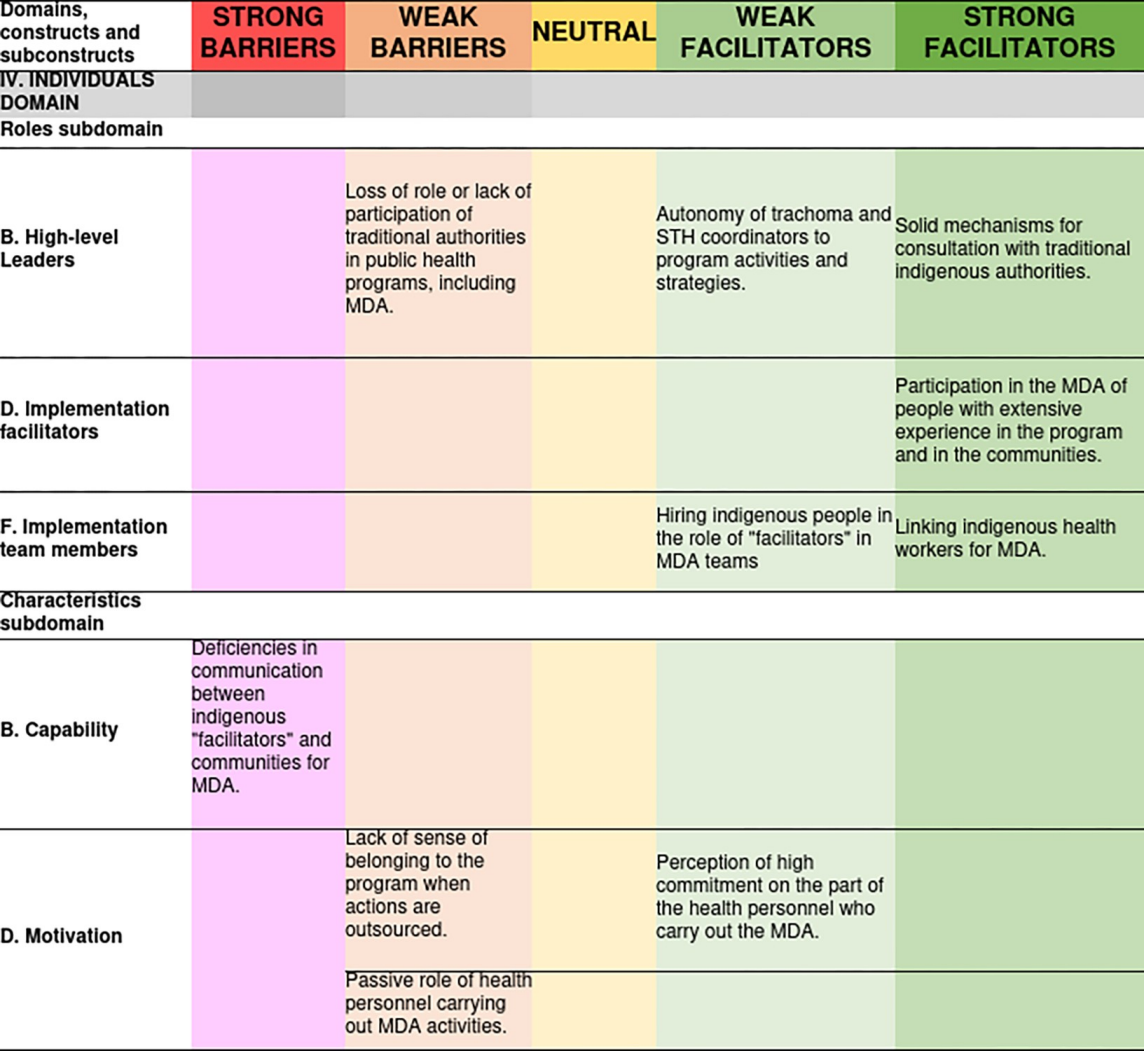
Access barriers and facilitators related to the individuals domain and its degree of influence on the MDA for trachoma and geohelminthiasis.

The MDA influencers related to this domain for each sub construct and their valence can be seen in Fig 5.

### Implementation process domain

Six constructs and four sub constructs of this CFIR domain were analyzed; one was adopted from the original CFIR (Adherence to Processes and Procedures for Implementation) since no affinity was found in the updated version [21]. Six strong barriers, six weak barriers, two neutral barriers, and one weak facilitator were identified.

Regarding the “Teaming” construct, the Department of Health for Amazonas has directly developed the MDA activities for trachoma. In the case of deworming, it was carried out with the local public hospital. The Municipal Health Secretariats did not participate in this activity, so they did not join efforts to improve MDA coverage.

In the "Assessing Needs” construct, "Innovation deliverers" sub construct, it was identified that the stated specific barrier (Lack of a prior verification process of supplies and drugs for MDA) referred to a single incident that did not have a more significant impact; therefore, its impact on MDA coverage was assessed as neutral.

When reviewing the emerging sub construct adopted from the original CFIR framework, called "Adherence to Processes and Procedures for Implementation", it was found that some members of the MDA teams had given drugs to people in transit from other countries who did not reside in the communities where the activity was being carried out, leaving local personnel who were eligible without drug. Likewise, it was identified that pregnant women in any trimester and breastfeeding were systematically excluded from mass drug administration with azithromycin.

The results of the review of the sub construct “Innovation Recipients” allowed the identification of missed opportunities and refusals to MDA in some households where the man was absent due to work and the woman, without prior permission from the man, did not accept the interventions of the health team. Low interest in health sector activities was also reported in some communities.

The “Assessing Context” construct reported that during the MDA development, there were barriers associated with cultural activities, rituals, festivities, and communication difficulties associated with language between the MDA team and the MDA beneficiaries. It was also mentioned that the interruption of the MDA because of the flow of tourists who came to specific river communities of the Amazon River made the population to prefer to sell them handicrafts and services instead of receiving medication.

Concerning the “Planning” construct of the MDA, 38% (60/159) of the study participants mentioned deficiencies in the dissemination and communication of the activities offered to the population in schools, colleges, and rural communities, mainly because they were not previously informed of the activity; 33% (52/159) mentioned that failures in complying with the schedules and agendas agreed upon in the consultation processes between the Department of Health for Amazonas, the hospital, and the indigenous leaders, which had a negative effect on the credibility of the health sector and the acceptance of the MDA.

*“… I am talking about what has happened in recent years. The contract is signed in the middle of the year, and the execution begins in September or October, so effectively, we do not get to carry out the two established rounds; in the time that goes from January to June, work tables are held, activities are carried out, the costing is made, it is checked whether the hospital agrees with the costs and so forth… in all that administrative process we are losing three or four months of fieldwork operations…”* [Translated quote from its original in Spanish] B13-P 10 Coo SDSA.

In the “Innovation Deliverers” sub construct, there was a high turnover of MDA personnel and a low supply of health personnel in the local market; short-term hiring, which was critical for people from the interior of the country, who could not afford to stay while waiting for new contracts; there were no guarantees of new hires in the future; the learning curve could not be overcome, and people with no experience or skills in working with ethnic groups always arrived.

“*There are few people as permanent staff. If they are hired, there are no attractive conditions or incentives that allow the staff to stay, so we try to strengthen the human talent that is specific to the territory, but still it is not enough”.* B14-P18 Dir. MSPS [Translated quote from its original in Spanish]

“Innovation Recipients” sub construct, it was mentioned that the adopted MDA implementation strategy had placed the community in a passive role, without them feeling that they were part of the solution to a health problem affecting them.

*“The Ticuna*, *the Yaguas*, *and the Bora ethnic groups hardly come*. *They are very shy*. *You have to go to their home instead of waiting for them to come*. *Thus*, *there must be someone in the community with authority to take ownership of the program and have good results*.*”* B15-P59 Puerto Nariño Leader. [Translated quote from its original in Spanish]

**Fig 6:**
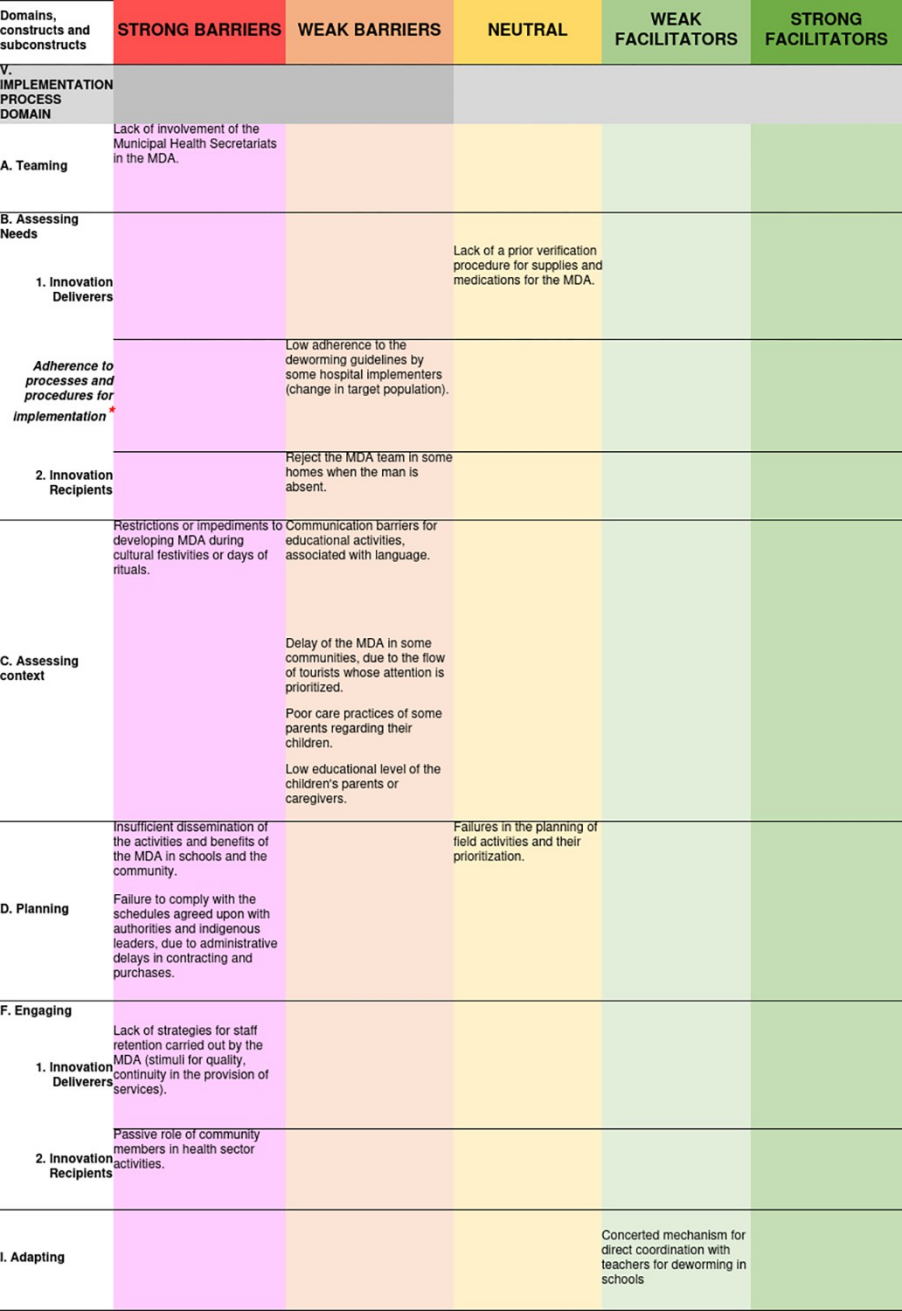
Access barriers and facilitators related to the implementation process domain and their degree of influence on MDA for trachoma and geohelminthiasis. *Construct taken from the original CFIR framework.

Finally, regarding the “Adapting” construct, it was identified that in order to simplify procedures between the health and education sectors and to solve the problems caused by communication breakdown, the geohelminthiasis program had agreed with the Amazonas Health Secretariate, the possibility of directly coordinating the deworming activity, the deworming strategy, and the procedure for parental consent with rectors and teachers. The barriers and facilitators related to this domain and their degree of influence on MDA can be seen in Fig 6.

## Discussion

The challenge of implementing the MDA strategies of azithromycin and albendazole for the elimination of trachoma and geohelminthiasis, respectively, in the context of the Amazon, went beyond geographical barriers and high operational costs of intervention. The study identified many more barriers to access to MDA than facilitators: 21 strong barriers, 30 weak, and six neutral; five weak and 11 strong facilitators. 62% (n = 13) of the strong barriers and 40% (n = 12) of the weak ones were concentrated in the “Outer setting domain”. On the other hand, only 16 facilitators were identified, 44% (n = 7) in the “Innovation” domain and 37% (n = 6) in the “Individuals” domain. This situation alerted the institutions responsible for the trachoma and geohelminthiasis elimination programs to identify those that were modifiable in order to generate an improvement plan to adjust processes and guidelines and make decisions to overcome, mitigate, or enhance them, as appropriate, aiming to achieve the MDA coverage goals.

Unlike other studies on access barriers to some health care analyzed under the conceptual framework of the original CFIR [23], it was considered relevant to detail and report the findings to the point of stating the specific barriers or facilitators within each domain, construct, and sub construct and to individually assess their degree of influence on coverage. It was believed that in this way, it was possible to concentrate on identifying and applying corrective measures more efficiently instead of simply reducing the complexity of each construct or sub construct to a single global assessment.

In terms of the design and operation of the SAFE and PCH strategies, the main difference identified was that the activities had been implemented independently of other public health programs in the former, whereas, in the second one, they had been integrated into the dynamics of the Collective Interventions Plan, which brought together activities of the other public health programs and strategies for communicable and noncommunicable diseases.

The implementer of the SAFE strategy was the Amazonas Health Secretariate, and that of the PCH strategy was the departmental public hospital. In both cases, community participation in the distribution of drugs was scarce and based on the logic of rounds or brigades of health workers teams who visited rural communities for one to three days, without fixed points for the MDA.

The main strengths identified were the prioritization of both events in the territorial public health agenda, the availability of economic resources for implementation, the presence of operational and administrative personnel with expertise in the subject and knowledge of the context, the coordination of interventions with indigenous leaders and local authorities, and the sensitivity and respect for the cultural differences of the target population, which translated into the sociocultural adaptation of the strategy, the latter also identified as a need in a study of geohelmintiasis carried out in Malawi [24]. For its part, community acceptance of the drug, was identified as the most important factor in a publication by Kuper et al, with contextual factors apparently not influencing the achievement of coverage above 80% [25].

Similar primary studies regarding access barriers and facilitators to MDA for trachoma, elaborated under the CFIR conceptual framework in the Americas or other regions of the world, were not found in the literature but in other regions and with other methodologies. A systematic review compiling findings from publications related to access barriers and facilitators that collected data on the four components of the SAFE strategy used the CFIR to guide the analysis [26]; in that study, the authors described high turnover of personnel, the hiring of health personnel who did not come from endemic areas, the lack of continuity of care in health services as the main structural barriers. They mentioned a reduction in MDA coverage when the health personnel “normalize unfavorable hygienic practices” or when personnel who delivered azithromycin felt demotivated because they felt they were not solving the structural problem: the lack of access to water. Moreover, the involvement of women who had undergone surgery for trachomatous trichiasis was mentioned as a facilitator of MDA in order to increase community participation.

In the case of geohelminthiasis, the study by Amy Roll and colleagues [24], conducted in three countries, identified a barrier shared in this study’s findings; it was related to the overload of activities of health personnel performing deworming activities, similar to what was experienced in the Amazon, given that the strategy was integrated with other public health programs.

Another study in Ethiopia identified the development of MDA campaigns at distant fixed stations for many families, the frequent side effects, rumors, lack of community and leadership involvement in the campaign, fasting, shortage of human personnel, and the lack of availability of short term supplies as barriers [27].

The difficulty in economic and logistical terms to develop in the Amazonas department a strategy of prior dissemination of the MDA campaign between three days and two weeks duration was highlighted, similar to those carried out in Ethiopia, with the involvement of political and religious leaders, health workers, women, and community representatives, and absence of other media with sufficient coverage. In this aspect became relevant to understand the magnitude of the negative impact of geographical access barriers and the high dispersion of the population of Amazonas.

Considering the local context, a "facilitator" named "ethnic liaison" was responsible for the prior dissemination of the MDA in the department of Amazonas, who at best-notified community leaders about the campaign to be developed but generally did it at the same time as the MDA. Information and education activities to prevent trachoma and geohelminthiasis were carried out simultaneously with the MDA; separating the time when the educator and the ethnic liaison of each group did their work from the rest of the members implied double logistics, which was impossible to finance in Amazonas.

Regarding the finding documented in another study of Ethiopia, by Asfaw et al, which evaluated access barriers to MDA for trachoma and geohelminthiasis, among others, weaknesses were shared in the social mobilization strategy not due to the quality of the contents but by the low prior dissemination of the workshops. It also empathized with the following barriers reported: a pattern of household dispersion and the mobile nature of semi-nomadic communities, erroneous beliefs related to drugs, lack of ownership of MDA strategies, unfavorable cultural and gender standards, and distrust in the government [28].

It was identified and included in the study a wide range of institutional actors from the health sector at the national, departmental, and institutional levels; from the education sector at the local level; local indigenous leaders; an agent of traditional medicine; and indigenous and multiracial beneficiaries of the programs in order to analyze the problem of interest from different perspectives, given its complexity.

The involvement of men and women was guaranteed in all spaces; however, in the intercultural dialogues with indigenous leaders it was proportionally lower because a low number of women occupied this role. The selection bias was reduced in the institutional participants of the health sector by convening all key institutions and all persons who met the inclusion criteria based on their role and influence on the MDA, that is, regardless of their ethnicity, sex, sexual identity, place of residence, political affiliation, etc., which guaranteed the heterogeneity of the respondents. All the convened agreed to participate voluntarily in our study.

A limitation was recognized regarding the representativeness of the agents of ancestral medicine in our study, given that only one midwife was included due to the difficulties of communication with the others. It was clarified that, in the Amazon, their work was not limited to attending pregnant women and newborns but, in general, to the health care of the population. Similarly, it was recognized a possible information bias associated with the lack of validation of the study results by some participants who no longer work in the institutions and the impossibility of doing so with non-institutional participants due to the high costs involved in visiting them a situation that mitigated by recording the sessions and reviewing the transcripts.

In general, various responses from participants suggested good acceptance of deworming in most communities and no interference or objection from ancestral physicians to perform it. Similarly, they reported a limitation of their ancestral medicine to treat trachoma, so they generally accepted azithromycin too.

Finally, given that one of the cross-cutting goals defined in the road map onneglected tropical diseases 2021–2030 [29] was to achieve 75% of coverage in integrated treatments of chemoprophylaxis and the fact that historically low coverage rates had been reported worldwide for azithromycin, the low number of research studies on implementation to assess the degree of preparedness of the institutions and the programs or health services, in the case of trachoma and geohelminthiasis, to implement those strategies and identify access barriers to MDA drew attention because it was believed that these studies were the best tool to understand the context and to adapt guidelines and strategies to achieve those goals.

## Conclusions

MDA coverage for trachoma and geohelminthiasis with azithromycin and albendazole or mebendazole in the department of Amazonas was influenced by an extensive list of access barriers, which exceeded that of facilitators. A priority must be to analyze each of them to identify strategies or actions to mitigate, eliminate, or enhance them, as applicable, and implement them for future MDA rounds, as well as its post-implementation evaluation.

## Supporting information

S1 Table Rating barriers and facilitators and degree of influence. (TIFF)

S2 Table Consolidated Criteria for Reporting Qualitative Research (COREQ) analysis. (DOCX)

S1 Fig Encoding tree. (TIF)
