# Supplementary figures and images for: Access barriers and facilitators to implement mass drugs administration strategies for eliminating trachoma and geohelminthiasis in the department of Amazonas, Colombia
Source: PLoS One. 2024 Dec 11;19(12):e0310143. doi: 10.1371/journal.pone.0310143 (PMC11633981; doi:10.1371/journal.pone.0310143)

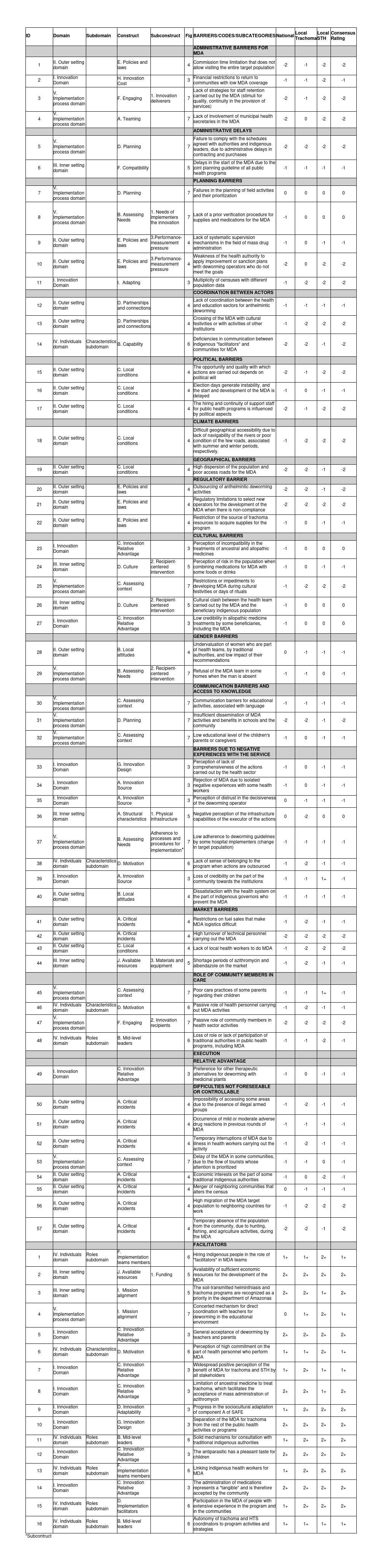

Supplement: S1 Table — (TIFF) [file pone.0310143.s001.tiff]

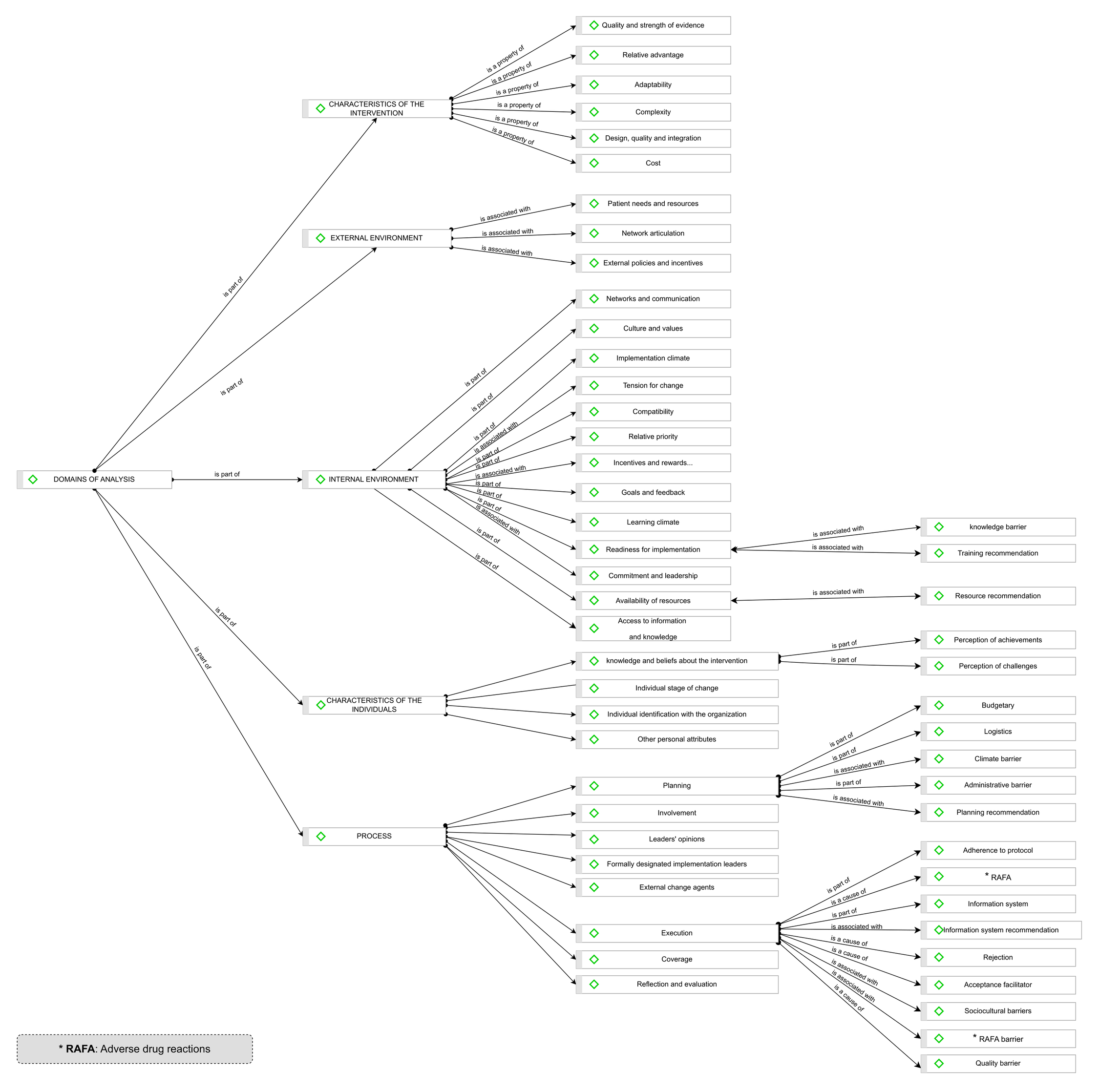

Supplement: S1 Fig — (TIF) [file pone.0310143.s003.tif]
