# Supplementary material for: Access barriers and facilitators to implement mass drugs administration strategies for eliminating trachoma and geohelminthiasis in the department of Amazonas, Colombia
Source: PLoS One. 2024 Dec 11;19(12):e0310143. doi: 10.1371/journal.pone.0310143 (PMC11633981; doi:10.1371/journal.pone.0310143)
Supplement: S2 Table — (DOCX) [file pone.0310143.s002.docx]

**Domain 1: Research team and reﬂexivity**

**Personal Characteristics**

| **No.** | **Item** | **Compliance description** |
| --- | --- | --- |
| **1** | Interviewer*/*facilitator | ***Which author/s conducted the interview or focus group?***  The contributions of the authors are described on page 1; the roles that participated in the direction of the methodologies are reported in lines 127-133 and 215-219. |
| **2** | Credentials | ***What were the researcher’s credentials? E.g. PhD, MD***  **WHO APPLIED QUALITATIVE METHODOLOGIES**  1. Bacteriologist, Sp in Quality Manager Systems, MSc Epidemiology  2. Anthropologist, MSc Gender  3. MD, Bacteriologist, MSc Public Health  4. Optometrist, MSc Public Health, PhD Public Health  *5.* Nourse, Sp Epidemiology  6. Optometrist, Sp Public health surveillance  *7.* Nutritionist  8.QFB, Sp, MSc Pharmacology, PhD Pharmacy  *9.* MD, Sp Epidemiology  10.Bacteriologist, Sp Epidemiology, Sp Health Economy, MpH.  11.Biologist, PhD Molecular Biology |
| **3** | Occupation | 1. National Coordinator of Emerging, Reemerging and NTD Group, MoH and Public Health and Epidemiology Chair Professor, UNAD.  2. Specialized professional contractor to support the sociocultural adaptation process of communicable disease programs, MoH  3. Researcher Professor, UNAD University.  4*.* National Dean School of Health Sciences, UNAD University  *5.* Public Health Surveillance Program Coordinator, Amazon Health Secretariat  6. Trachoma Program Coordinator, Amazon Health Secretariat  7*.* Geohelminthiasis Program Coordinator, Amazon Health Secretariat  *8.* Director of IFARMA foundation, Bogotá  *9.* Senior Advisor of Ifarma foundation, Bogotá  10. Specialized professional contractor, coordinator of the National Acute Respiratory Infection and Acute Diarrheal Disease Program, MoH.  11. Researcher Professor, UNAD University, Bioinnova Research Group Director |
| **4** | Geneder | Interviews and focus groups were conducted by a man and a woman, with rotating roles; intercultural dialogues were conducted by a woman. |
| **5** | Experience and training | 1. Work experience 28 years, public health experience of 20 years:   Insitucional level: Bactriologist of the analytical area, Jorge Piñeros Corpas Clinic, among others 6 years.  Department level: Public health programs (Communicable diseases and public health laboratory), 8 years.  National Level: Public health programs at the national level – National Coordinator at the Ministry of Health (12 years, communicable diseases- Neglected tropical diseases).  Supra National level as consultant: Tuberculosis (PAHO/WHO), Malaria (ORAS-CONHU).  10 years of experience in public health work with indigenous people in Guainía and Vaupés and with other specific public health programs or projects in others indigenous communities of Colombian departments.  Trainings  Epidemiology for health managers, based on the internet. Johns Hopkins Bloomberg School. Mexico D.F  SORT-IT operations research and Public Health Problem Solving (Johns Hopkins Bloomberg School), active researcher in neglected tropical diseases with more than 10 publications including qualitative and quantitative analyses.  Implementation research, online training, WHO.  Master's grader in trachoma diagnosis (Tropical Data/WHO)  Co-director of a master's thesis in epidemiology at Antioquia University of in Implementation Research in neglected tropical diseases.  Memberships  Active member of the Bioinnova research group, and researcher attached to the Ministry of Science, Technology and Innovation,  Honorary and lifetime member, Colombian Association of Public Health,  Honorary and lifetime member of the Colombian Society for the History of Medicine.  2. Anthropologist graduated from the National University of Colombia and master in gender and politics from the Latin American Faculty of Social Sciences of Argentina.15 years of work experience in the public and private 15 years in the public and private sector, with field work in the departments of Casanare, Tolima, Guainía, Vaupés, La Guajira, Antioquia, Amazonas and Cundinamarca, in Colombia.  Experience working in Bolivia with indigenous organizations. Interested in issues of health promotion, gender, violence, public policies and community development.  Research experience with people in conditions of high vulnerability, peasants, indigenous and Afro-Colombian communities, indigenous and Afro-Colombian communities, pregnant adolescents, people deprived of liberty and victims of the and victims of the Colombian armed conflict. Knowledge and skills in the implementation of qualitative research techniques such as ethnography, individual and group interviews, focus groups and individual and group interviews, focus groups, open meetings, consultations, consultancies and workshops, among others.  Development of the community IMCI component: strengthening families and communities for comprehensive early childhood health care. University of the Andes.  Pan American Health Organization -PAHO-. August 2016.  Editing and academic writing with emphasis on evaluation of scientific articles. University de la Sabana and Latin American Foundation for the Promotion of Science. 2008.  Memberships  Research Group in Pediatrics. Department of Pediatrics, School of Medicine, National University of Colombia.  Health Policy Research Group. Faculty of Medicine. National University of Colombia.   1. María Consuelo Bernal Lizarazú   Associate Professor of the School of Health Sciences of the National Open and Distance University -UNAD, medical professional with a master's degree in Public Health and Social Development and a master's degree in Microbiology, emphasis on parasitology. More than 20 years of experience in the design, planning and execution of research projects in epidemiology, parasitology and public health, as well as an active member of the UNAD Research Ethics Committee. Junior Researcher at the Ministry of Science, Technology and Innovation of Colombia and active member of the Bioinnova research group  .   1. Myriam Leonor Torres   5. Public Health Surveillance Manager and Epidemiology of the Amazon Health Department, with experience in planning and executing projects in public health, as well as in the technical and financial management of resources for public health. Administrative experience and in field work related to the care of outbreaks and epidemics, especially related to communicable diseases; responsible for the notification of events of interest in public health in SIVIGILA, such as trachoma, malaria, dengue, tuberculosis, among others.  6. Carol Viviana Araque Sarmiento, She will perform functions as a co-investigator participating in the training, organization and supervision of a research team. field work, evaluation of patients with trachoma and follow-up of surgical patients, consolidation and debugging of databases and publications; she is an optometrist, master of public health, specialization student in public health surveillance, reference of the Trachoma Elimination Program in the Amazon Health Secretariat, with operational and administrative experience in it.  7. Sonia Pulido  8. Postdoctoral stays Personalized medicines as an alternative to guarantee access to medicines and their proper use in the Colombian health system.  Position: Analysis and updating of the Pharmaceutical Policy of the Services Unit of ECOPETROL S.A. November 2010 January 2011 - Other - Position: Evaluation of 21 health technologies (medications) for their inclusion in the Colombian Mandatory Health Plan for the year 2011. November 2010 F from 2011 - Other - Position: Update of the List of Essential Medicines of the Health Services Unit of ECOPTEROL S.A. September 2007 June 2008 - Specialized Technical Service - Position: Systematic search for information and preparation of technical concepts on drugs for the Scientific Technical Committee of the Health Services Unit of Ecopetrol S.A. August 2007 November 2011.  Administration activities - Specialized Technical Service –  Position: Evaluation of the national pharmaceutical policy and its development in the district and design of the district pharmaceutical policy. February 2011 June 2011 - Specialized Technical Service - Position: Comprehensive technical support in the operation of the University's Drug Information Center (CIMUN) and maintenance databases of the Network for the Rational Use of Medicines (RAM) research group. September 2009 February 2010 teaching activities - Teaching/Undergraduate Teaching - Name of the course: Hospital Pharmacy, September 25, 2010 October 2010 - Teaching/Undergraduate Teaching - Course name: Hospital Pharmacy, August 30, 2008 June 2009 research activities - Research and Development - Title: Surveillance of adverse events associated with Tramadol, Dipyrone, Warfarin and Heparin - WHEDIT February 2008 May 2008 - Research and Development - Title: Design and development of a Pharmacotherapeutic Follow-up method for patients with Epilepsy June 2008  Lines of investigation Access to medications, Active: Promotion of the Rational Use of Medicines.  Scholarship program for PhD abroad 2011, - November 2011 SCHOLARSHIP PROGRAM FOR OUTSTANDING POSTGRADUATE STUDENTS, NATIONAL UNIVERSITY OF COLOMBIA - February 2008; Director or tutor of master's and doctoral theses at the National University of Colombia  9. Francisco Augusto Rossi  Member of the National Medicines Commission, adviser to the Social Security Council in Health during the year 2001. Special participation for the regulation of the incorporation of the drug component in the reform of the health system - Social Security System in Health, Law 100 of 1993, and for the creation of INVIMA, Agency Regulatory for Medicines, Food and Health Technology. Official of the Pan American Organization of the Health in Bolivia and Colombia for more than 10 years. Temporary consultant for organizations such as the Organization World Health Organization, the Pan American Health Organization, the Inter-American Development Bank, the Bank World Cup and the European Union, in various countries such as Equatorial Guinea, Argentina, Bolivia, Paraguay, Nicaragua, Ecuador, Peru, the Dominican Republic, Guatemala and Honduras. Adviser to the Ministry of Health of Colombia for the Pharmaceutical Policy and for the negotiations of the Free Trade Agreement with the United States of America until January 2004. Coordinator of the Intellectual Property Project and access to medicines of the Program of the United Nations Development Program, UNDP, located at the UNDP International Poverty Center in Brasilia from February 2004 to March 2007. Currently Senior Advisor of the la Fundación Instituto para la investigación de medicamentos en los sistemas de Salud, IFARMA,in Bogotá.  10 Luz Mery Bernal Parra  Associate Professor of the School of Health Sciences of the National Open and Distance University -UNAD, biologist, with a master's degree in biology, emphasis on genetics and PhD. in biological sciences, emphasis in human genetics. With experience in the design, planning and execution of research projects related to the comprehensive approach to different communicable and non-communicable diseases, with a molecular diagnostic approach and its interaction with public health. Technical management of UNAD research processes. Director of the Bioinnova research group and junior researcher at the Ministry of Science, Technology and Innovation of Colombia. |
| **6** | Relationship established | ***Was a relationship established prior to study commencement?***  Several of the co-authors work on a daily basis with different public health, epidemiology and academic topics and we know each other prior to the development of this work. |
| **7** | Participant knowledge of the interviewer | ***What did the participants know about the researcher? e.g. personal goals, reasons for doing the research***  The researchers were introduced to the participants by local health personnel; After the presentation, the objectives, benefits and risks of the study were explained, in accordance with the ethical standards of research in Colombia. Lines 255-256 |
| **8** | Interviewer characteristics | ***What characteristics were reported about the interviewer/facilitator? e.g. Bias, assumptions, reasons and interests in the research topic***  No conflict of interest was declared by the authors; The limitations of the study were described in the manuscript, lines 665-673. |

**Domain 2: Study design**

**Theoretical framework**

| **9** | Methodological orientation and Theory | ***What methodological orientation was stated to underpin the study?***  **Content analysis** |
| --- | --- | --- |
| Participant selection | | |
| **10** | Sampling | **How were participants selected? *e.g. purposive, convenience, consecutive, snowball***  The selection criteria of the participants are described in the methodology (lines 143-174) |
| **11** | How were participants approached? e*.g. face-to-face, telephone, mail, email* | **Method of approach**  **Approach methods were face to face, described in the methods section (lines 121-126 and 194-226)** |
| **12** | Sample size | **How many participants were in the study?**  Lines 144-162 ; 258-270 and Fig 1. |
| **13** | Non-participation | ***How many people refused to participate or dropped out? Reasons***  All participants accepted the invitation to the study 268-269 |
| Setting | | |
| **14** | Setting of data collection | ***Where was the data collected? e.g. home, clinic, workplace***  Reported lines 213-214 |
| **15** | Presence of non-participants | ***Was anyone else present besides the participants and researchers?***  Reported: lines 130-132 |
| **16** | Description of sample | ***What are the important characteristics of the sample? e.g. demographic data, date***  Reported lines: 146-162 |
| Data collection | | |
| **17** | Interview guide | ***Were questions, prompts, guides provided by the authors? Was it pilot tested?***  Reported lines 175-192 |
| **18** | Repeat interviews | **Were repeat interviews carried out? If yes, how many?**  Reported Lines 195-214; line 272 Fig 1 |
| **19** | Audio*/*visual recording | ***Did the research use audio or visual recording to collect the data?***  Reported: line 228 |
| **20** | Field notes | ***Were ﬁeld notes made during and/or after the interview or focus group?***  216-217 |
| **21** | Duration | ***What was the duration of the interviews or focus group?***  Reported lines 226-228 |
| **22** | Data saturation | ***Was data saturation discussed?***  Reported lines: 153-154, 157, 224-226 |
| **23** | Transcripts returned | Were transcripts returned to participants for comment and*/*or correction?  Lines 671-675 |

**Domain 3: Analysis and ﬁndings**

**Data analysis**

| **24** | Number of data coders | ***How many data coders coded the data?***  236 |
| --- | --- | --- |
| **25** | Description of the coding tree | ***Did authors provide a description of the coding tree?***  239 -240 and S1Fig |
| **26** | Derivation of themes | ***Were themes identiﬁed in advance or derived from the data?***  A mixed method was used. Texts were coded using the combination of the indictive and deductive methods.  Reported: Lines 176-192; 239-240 |
| **27** | Software | ***What software, if applicable, was used to manage the data?***  Reported Line 235 |
| **28** | Participant checking | ***Did participants provide feedback on the ﬁndings?***  Reported 250-252 and we reported as a limitation in some participants: Lines 672 - 676 and we report the way we mitigate possible bias. |

**Reporting**

| **29** | Quotations presented | ***Were participant quotations presented to illustrate the themes / ﬁndings? Was each quotation identiﬁed? e.g. participant number***  The voice of the participants was incorporated into 15 textual quotes in the results, pages: 13,14,16,17,18,19,21,23 and 24  the appointments were clearly identified with the role of the participant and a codification of the institution to which they belong |
| --- | --- | --- |
| **30** | Data and ﬁndings consistent | ***Was there consistency between the data presented and the ﬁndings?***  We report the access barriers and facilitators that emerged from the interviews, focus groups and intercultural dialogues (our study objectives); these barriers and facilitators were matched with the CFIR constructs and on the joint analysis of the construct and individual of each barrier or facilitator, the results were presented and the discussion was written with them |
| **31** | Clarity of major themes | ***Were major themes clearly presented in the ﬁndings?***  The major findings were presented in figures 3, 4, 5, 6 and 7  They are color coded depending on whether they correspond to a barrier or a facilitator and the degree of influence (neutral, weak or strong). |
| **32** | Clarity of minor themes | ***Is there a description of diverse cases or discussion of minor themes?***  Minor themes are discussed in more detail through quotations and interpretations under major theme headings in the results section |
